# Functional analysis of *Girardia tigrina* transcriptome seeds pipeline for anthelmintic target discovery

**DOI:** 10.1186/s13071-014-0622-3

**Published:** 2015-01-20

**Authors:** Nicolas J Wheeler, Prince N Agbedanu, Michael J Kimber, Paula Ribeiro, Tim A Day, Mostafa Zamanian

**Affiliations:** Department of Biomedical Sciences, Iowa State University, Ames, IA 50010 USA; Institute of Parasitology, McGill University, Ste. Anne de Bellevue, QC H9X 3V9 Canada

**Keywords:** Schistosomiasis, *Girardia* tigrina, Planaria, RNA-Seq, Drug discovery

## Abstract

**Background:**

Neglected diseases caused by helminth infections impose a massive hindrance to progress in the developing world. While basic research on parasitic flatworms (platyhelminths) continues to expand, researchers have yet to broadly adopt a free-living model to complement the study of these important parasites.

**Methods:**

We report the high-coverage sequencing (RNA-Seq) and assembly of the transcriptome of the planarian *Girardia tigrina* across a set of dynamic conditions. The assembly was annotated and extensive orthology analysis was used to seed a pipeline for the rational prioritization and validation of putative anthelmintic targets. A small number of targets conserved between parasitic and free-living flatworms were comparatively interrogated.

**Results:**

240 million paired-end reads were assembled *de novo* to produce a strictly filtered predicted proteome consisting of over 22,000 proteins. Gene Ontology annotations were extended to 16,467 proteins. 2,693 sequences were identified in orthology groups spanning flukes, tapeworms and planaria, with 441 highlighted as belonging to druggable protein families. Chemical inhibitors were used on three targets in pharmacological screens using both planaria and schistosomula, revealing distinct motility phenotypes that were shown to correlate with planarian RNAi phenotypes.

**Conclusions:**

This work provides the first comprehensive and annotated sequence resource for the model planarian *G. tigrina*, alongside a prioritized list of candidate drug targets conserved among parasitic and free-living flatworms. As proof of principle, we show that a simple RNAi and pharmacology pipeline in the more convenient planarian model system can inform parasite biology and serve as an efficient screening tool for the identification of lucrative anthelmintic targets.

**Electronic supplementary material:**

The online version of this article (doi:10.1186/s13071-014-0622-3) contains supplementary material, which is available to authorized users.

## Background

The Platyhelminthes (flatworms) comprise a diverse phylum of medically and economically important species. Trematodes (flukes) and cestodes (tapeworms) are the etiological agents of several Neglected Tropical Diseases (NTDs) that disproportionately devastate the health and economic prospects of the poor across much of the developing world. Schistosomes infect over 220 million in sub-Saharan Africa alone, and 600–800 million live at risk of infection worldwide [1]. Echinococcosis and cysticercosis, while less prevalent than schistosomiasis, are zoonotic parasitic diseases of great public health importance. These neglected diseases inflict significant morbidity and mortality, accounting for upwards of 280,000 deaths [2] and an annual loss of between 3.5 - 70 million disability-adjusted life years (DALYs) [3].

The prioritization of flatworm-associated NTDs by the World Health Organization [4] underscores the urgency of efforts to control infection and to develop new anthelmintic treatments. The threat of drug resistance [5] further calls attention to the need for novel pipelines for drug target validation and drug discovery [6]. Against this backdrop, free-living flatworms represent a new and potentially powerful screening model for parasite drug discovery efforts [7]. Members of the free-living genus *Planaria* are widely interrogated in the realm of stem cell biology due to their remarkable regenerative abilities [8]. In comparison to their parasitic counterparts, planarians are much more amenable to modern genetic protocols and their culture and maintenance within the laboratory is relatively inexpensive and simple. Many behavioral, biochemical, and morphological phenotypes have also been described for planaria [9-11], enabling straightforward inferences of function from combined reverse genetic, pharmacological, and phenotypic analyses.

In the case of schistosomes, it is necessary to maintain active populations of freshwater snails as intermediate hosts, manage periodic shedding of the infective cercariae, induce transformation to schistosomula or allow for penetrance into a definitive host (usually mice). The process is difficult, time consuming, moderately dangerous, and, for many labs, cost prohibitive. These concerns underpin efforts to extend the utility of planarian biology to the study of nearly-related parasites [12], mirroring the important role that *Caenorhabditis elegans* has played in furthering our understanding of the biology of parasitic nematodes [13].

A number of planarian species see use in the laboratory, with varying modes of reproduction, regenerative potential, and genome ploidy. *Schmidtea mediterranea* is among the most widely studied species. Clonal lines of *S. mediterranea* have been propagated to mitigate genetic heterogeneity, and both genomic [14] and transcriptomic [15] data have been published for this stable diploid [16-18]. Other notable planarian species include *Girardia* (formerly *Dugesia*) *tigrina* and *Dugesia japonica* [19]. Genome assembly and analyses are partly complicated in these species due to their mixoploid genomes and the presence of large numbers of transposable elements [20]. No significant sequence resources yet exist for *G. tigrina*, despite the convenient commercial availability of this species and its broad adoption in the fields of regeneration [21], pharmacology [22] and learning and memory [23].

The emergence of a comprehensive sequence resource for *G. tigrina* will open avenues for more precise biological manipulation of these planaria. RNA-Seq provides a powerful platform for producing a high-coverage transcriptome, without the complications of whole genome assembly. The selection of *G. tigrina* for this undertaking presents a reasonable trade-off, whereby some level of genetic heterogeneity is accepted for the greater ease of procuring, maintaining and scaling colonies, in comparison to clonally-derived laboratory strains. Genetic variation within this sexual strain is minor with respect to the accepted genetic distance between planaria and the flatworm parasites for which they are to serve as models. Although computationally intensive in the absence of a reference genome, a high-quality de novo transcriptome assembly would allow for closer examination of our overarching hypothesis that *G. tigrina* could provide a shortcut to identifying potential drug targets in the phylum.

## Methods

### Planarian culture and RNA isolation

*G. tigrina* (Ward’s Natural Science, Rochester, NY) colonies were maintained in aerated spring water. Planarians were starved for one week prior to RNA isolation. Five animals were randomly selected per experimental condition on day 7. Each group was washed repeatedly with spring water and tissue grinding was carried out using mortar and pestle in the presence of liquid nitrogen. A hybrid TRIzol (Invitrogen)/ RNeasy (Qiagen) protocol was used to isolate total RNA from ground tissue, whereby supernatants from the chloroform phase separation were combined with an equal volume of 100% ethanol and loaded into RNeasy columns for purification. Total RNA quality and concentration was assessed with an Agilent Bioanalyzer 2100. RNA integrity number (RIN) proved to be a poor benchmark of RNA quality, as the *G. tigrina* 28S rRNA subunit is evidently converted into fragments that co-migrate with 18S rRNA to produce a triple-peak, giving the misleading appearance of RNA degradation. All samples yielded at least 1 ug/ul of RNA when eluted in 40 ul of H20, with an OD A260/A280 of ∼ 2.1 and OD A260/A230 of ∼ 2.2.

### Library preparation and Illumina paired-end RNA-seq

Illumina HiSeq 2000 paired-end (2x100 bp) library preparation and sequencing was carried out at the McGill University and Genome Quebec Innovation Center. The four RNA samples were multiplexed across two sequencing lanes with an average fragment size of ∼ 350 bp, corresponding to an average insert size of ∼ 225 bp. The sequencer run yielded ∼ 30 million paired-end reads per sample (241 million total paired-end reads) with an average Phred quality score of 37.

### De novo transcriptome assembly

Adapter sequences were trimmed and reads were passed through a sliding window quality filter (window size = 4, minimum average quality score = 25) using Trimmomatic 0.22 [24]. Paired-end reads and singletons ≥ 50 bp in length were retained. Overlapping paired-end reads were identified and merged using FLASH [25] with an expected insert size of 220 bp. Quality control and read collapsing led to a total filtered pool of 165 million paired-end reads and 55 million singletons. Surviving reads were combined and fed into the Trinity [26] pipeline for *de novo* assembly, performed on the GLUMEQ Guillimin supercomputer maintained by McGill University. Assembly optimization and runs were carried out on a 1 TB ScaleMP node that allows for a virtualized shared large memory environment required by the OpenMP standard. Final assembly was carried out with a minimum k-mer coverage of 2 and the default k-mer size of 25. Complex graphs that proved unresolvable within a 6 hour window were manually excised to allow the assembly to proceed. Separately, an available Python script was used to feed the same read pool into the Velvet [27] pipeline and to generate multiple k-mer assemblies (k = 21, 25, 29 and 33) for merging with Oases (k = 25) [28]. The minimum contig or transcript length for both assembly pipelines was set to 200 nt. The statistical software R [29] was used to generate and evaluate assembly statistics. Further bioinformatic analysis was restricted to the Trinity transcriptome.

### Transcriptome filtering and annotation

Filtered paired-end reads were mapped to the Trinity transcriptome with Bowtie [30]. Abundance estimation with RSEM [31] was used to select for transcripts that accounted for at least 1% of the per-component (IsoPct) expression and that met a TPM cutoff of 1. Open reading frames (ORFs) with coding potential were predicted from the final transcriptome using log-likelihood scores based on codon usage with Transdecoder (http://transdecoder.sourceforge.net/). The resulting predicted proteome was further filtered with CD-HIT-EST [32] at a threshold of 0.95. BLAST2GO [33] was used to functionally annotate the *G. tigrina* predicted proteome and to assign GO terms to predicted proteins.

### Differential expression analysis

Condition-specific abundance estimation was carried out with Bowtie and RSEM using the final filtered transcriptome. Existing Trinity scripts and the R/Bioconductor packages DESeq and edgeR of the statistical programming language R were used to identify differentially expressed transcripts. A threshold e-value of 10–3 and minimum four-fold expression changes were used to select and cluster transcripts as either up or down-regulated. Transcript sets were then mapped to previous annotations, where available.

### Orthology analysis and drug target prioritization

Proteinortho [34] was used to detect ortholog groups between and among the *G. tigrina*, *S. mansoni* and *E. multilocularis* predicted proteomes, with default parameters and a coverage threshold of 0.5. The predicted parasite proteins were downloaded from GeneDB [35]. The visualization tool Circos [36] was utilized to organize and display the orthologous relationships among these species in the context of Trematoda and Cestoda synteny. Provided that a transcriptome does not imply any spatial or chromosomal arrangement, the *G. tigrina* transcripts were arbitrarily arranged on a pseudo-chromosome - designated as an ideogram - to enable visualization. GFF files obtained from GeneDB containing gene coordinate data for the two parasitic species were parsed for necessary sequence features using a set of in-house Python scripts and used to draw orthologous “links” between ideograms. Heatmap data was created by running command-line BLAST [37]. Similarity calculations were carried out with *G. tigrina* orthologs as queries against their corresponding parasitic orthologs, as well as with parasitic orthologs as queries against the RefSeq human proteome [38]. Sequence homology was used to select orthologs that displayed high sequence similarity among the three examined flatworm species, as well those sufficiently diverged from their nearest-identifiable human homolog. These prioritized ortholog groups were mined with GO IDs, along with manually-selected ID’s, to extract and highlight annotated sequences belonging to notoriously druggable protein families [39].

### Parasite maintenance

Snails (*Biomphalaria glabrata*) infected with *S. mansoni* were provided by the Biomedical Research Institute (BRI) (Rockville, MD). Cercariae were shed from snails by light exposure and subsequently mechanically transformed to schistosomula *in vitro* per existing protocols [40]. Somules were cultured in modified Basch medium (containing 10% Fetal Bovine Serum) at 37°C in 5% CO2 atmosphere.

### Schistosomula assays

Three small molecule inhibitors (CK-666, ARPC2; 3-NPA, SDH1; rotenone, NDUFV2) were purchased from Sigma-Aldrich (St. Louis, MO). Newly transformed schistosomula were incubated with various concentrations of each inhibitor for 30 minutes and recorded at 10× magnification for 5 minutes. Approximately 200 worms were assayed per experimental well, and 12 were randomly selected for video capture and analysis. The wrMTrck plugin of ImageJ [41] was used to track schistosomules and quantify motility in terms of contractile rate (body bends per second; BPS).

### Planarian pharmacology and motility analysis

*G. tigrina* individuals were placed in 35 mm dishes filled with 4 mL of media supplemented with inhibitor at varying concentrations or an equal amount of solvent control, and the dishes were placed on a light box in a dark room. After 30 minutes of incubation, worms were recorded for 5 minutes by EthoVision [42], and motility was quantified by dividing the parameter DistanceTraveled (mm) by TimeInZone (s). Down-sampling was set to 5 to ensure that small bending and twisting motions were not factored. Tracking profiles were visually diagnosed for errors and manually edited where required. Errors were most often attributable to light reflections off of the surface of liquid media or imperfect arena definitions.

### Planarian RNAi and regenerative assays

Total RNA was extracted from homogenized *G. tigrina* and converted to cDNA with Ambion’s RetroScript RT kit. 600 bp sequences were PCR amplified using primers designed with Primer3 [43]. T7 promoter sites were added using a two-step PCR protocol, and dsRNA was transcribed with the Ambion MegaScript RNAi kit. dsRNA was added directly to homogenized liver paste according to prescribed methods [44] (10 umol/worm for one hour). Feedings were performed on Days 1, 3, and 5, and worms were bisected immediately above the pharynx on Day 6. A minimum of 10 planaria (20 worm halves) were used for each experiment. Two worms from each experimental group were set aside for semi-quantitative RT-PCR performed with Ambion’s QuantumRNA18S Internal Standards kit. In each experiment, 10 planaria (20 worm-halves) were observed for defects in regeneration over the full regeneration period (∼2-3 weeks).

## Results and discussion

### De novo transcriptome assembly

**Figure 1:**
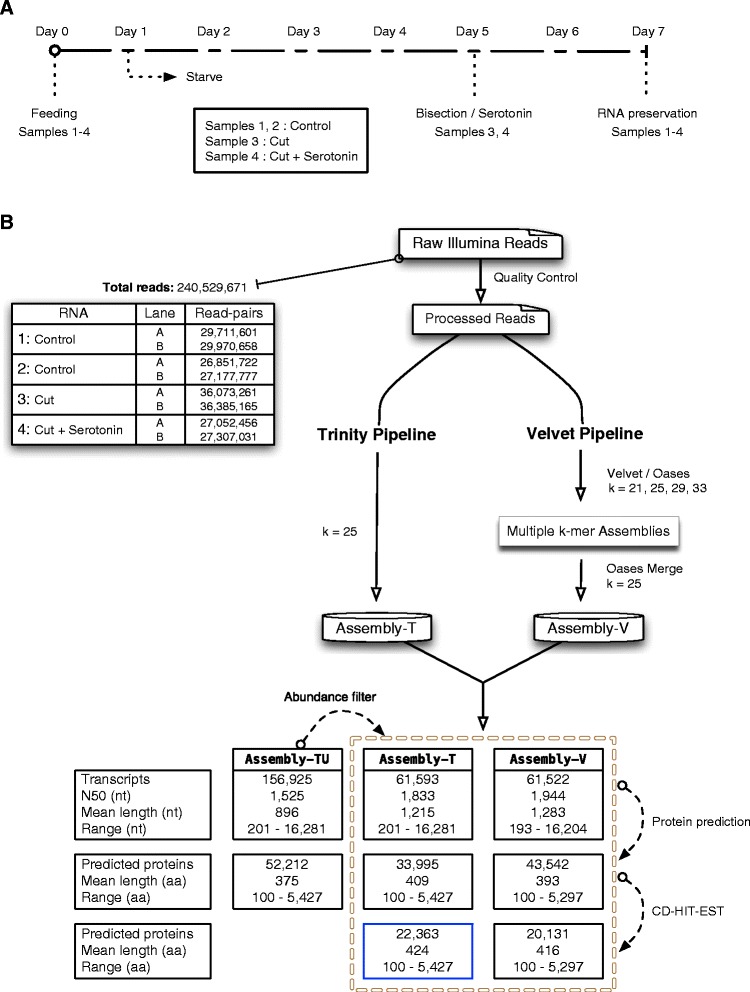
**RNA-Seq workflow. A)** Planarian feed-starve cycle and RNA extraction timeline. **B)** Processing of raw reads and parallel de novo transcriptome assembly using the Trinity and Velvet/Oases pipelines. The table depicts relevant statistics for the transcriptomes and predicted proteomes associated with each pipeline through various post-assembly filtering stages.

To improve the odds of comprehensive transcript capture, RNA was isolated from *G. tigrina* across a set of dynamic conditions. Planaria were passaged through a feed-starve cycle under different conditions prior to RNA extraction (Figure 1A). Worms were left untreated, cut transversely, and cut tranversely while incubated in the presence of the biogenic amine serotonin (5-hydroxytryptamine: 5HT). The aim of bisecting planaria was to elicit activation of potentially dormant regeneration- associated transcripts [45]. Serotonin was included due to its abundance and wide distribution in flatworm nervous systems, as well as the fundamental role it plays in parasite neuromuscular signaling [46].

**Figure 2:**
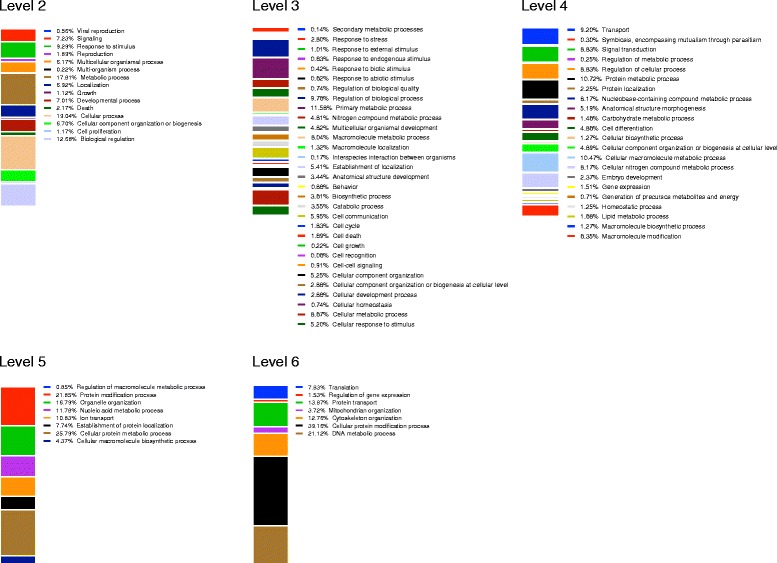
**Comprehensive annotation of RNA-Seq transcripts.** GO annotations reveal the wide range of biological process associated with the *G. tigrina* predicted proteome. Level 2 categories provide the most general functional annotation, with the largest fraction of proteins assigned to cellular and metabolic processes. The majority of signal transduction related proteins are captured in Level 4 annotations, and Level 6 annotations contain predictions for smaller numbers of proteins implicated in more specialized pathways (e.g. protein modification and DNA metabolism).

Total RNA was extracted and assessed for quality in preparation for Illumina paired-end (2×100 bp) RNA sequence (RNA-Seq). Read sets were combined for adapter-trimming, quality control and *de novo* assembly using two independent pipelines. Trinity [26] was used alongside a multiple k-mer (k = 21, 25, 29 and 33) Velvet/Oases [27,28] pipeline to produce initial assemblies, as depicted in Figure 2B. The Trinity assembly was filtered at a low abundance threshold after transcript abundance estimation vis RSEM [31]. The final Trinity (Assembly-T) and Velvet (Assembly-V) assemblies exhibit similar statistical profiles, with a comparable total transcript count, mean transcript length, N50, and transcript length range (Figure 1B). Both assemblies compare very favorably to other published planarian assemblies, due in part to the large read count and the computationally expensive incorporation of all available reads [15,16,19].

### Protein prediction and transcriptome annotation

Predicted proteomes were created for each assembly using Transdecoder (http://transdecoder.sourceforge.net/) to evaluate the coding potential of open reading frames based on codon usage. After subtraction of redundant proteins, the Assembly-T proteome had a marginally larger unique protein count and was therefore used for all subsequent analysis. The 22,363 predicted proteins from this dataset were used in blastx [37] queries against the NCBI nr database. 16,467 sequences had at least one significant hit (e-value < 0.001) and the top 20 hits were retained for each sequence. We then applied Gene Ontology (GO) annotations to the *de novo* proteome using the Blast2GO [33] pipeline. The mapping of sequence-specific blast results to GO identifiers resulted in functional annotation of all 16,467 proteins with significant blast hits, accounting for 73% of the predicted proteome. This was complemented by InterProScan [47] domain mapping, resulting in the identification of at least one domain for 18,051 protein sequences.

Annotated predicted proteins were categorized according to their involvements in various biological processes at different hierarchy levels. Figure 2 depicts this categorization from more general level 2 categories through more specific level 6 categories, with many proteins binned into multiple categories. Separately, to further gauge coverage of core pathways, Kegg [48] pathway mapping was carried out. As a representative example, over 95% of core reference pathway enzymes were identified (data not shown). The very small numbers of unmapped enzymes in these and related metabolic pathways could result partly from fundamental biological differences, as opposed to gaps in our dataset.

### Identification of differentially expressed transcripts

While the inclusion of different treatment conditions was primarily aimed at increasing transcript capture, it also presents an opportunity to identify transcripts that are significantly upregulated or downregulated with respect to these conditions. Previous investigators have carried out experiments to identify regeneration-associated genes in *S. mediterranea* [11] by performing RNAi screens to perturb normal regeneration. While many transcripts show greater than four-fold differences in expression between control and cut worms (e.g. RNA-dependent RNA polymerase, RNA helicases, reverse transcriptase), these data do not lend themselves to facile implications of molecular mechanisms of regeneration and are provided for further examination and investigation (Additional file 1).

### Identification of planarian-parasite orthologs

**Figure 3:**
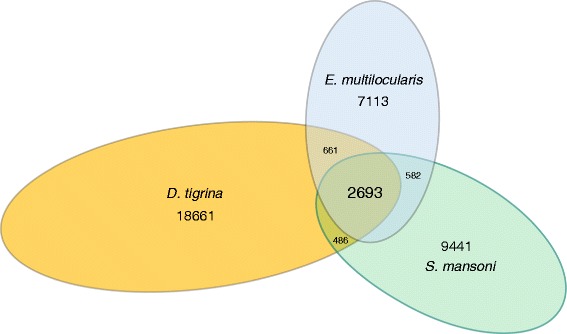
**Protein-level orthology among free-living and parasitic flatworms.** Orthology analysis via Proteinortho identified 2,693 orthologues shared among all 3 flatworms, and several hundred between flatworm pairs. In this high-stringency approach, the majority of *G. tigrina* transcripts were not identified as bona fide orthologs, despite substantial sequence homology.

The sequencing and assembly of the *G. tigrina* transcriptome allows for an important genetic comparison between this free-living worm and its pathogenic relatives. Proteinortho [34] was used to identify orthologous protein sequence groups shared between and among *G. tigrina* and the parasitic species *Schistosoma mansoni* and *Echinococcosis multilocularis*. This program employs an efficient reciprocal best alignment algorithm, yielding a very conservative but reliable subset of likely ortholog groups using the predicted proteomes of a set of species. A total of 3,179 orthologs were identified for the *G. tigrina*- *S. mansoni* pairing, contrasted with a more expansive pairwise homology (BLASTp) search which identifies over 10,000 significant (E-value > 0.01) hits. Overall, 2,693 sequences were identified as belonging to ortholog groups that spanned all three species (Figure 3).

**Figure 4:**
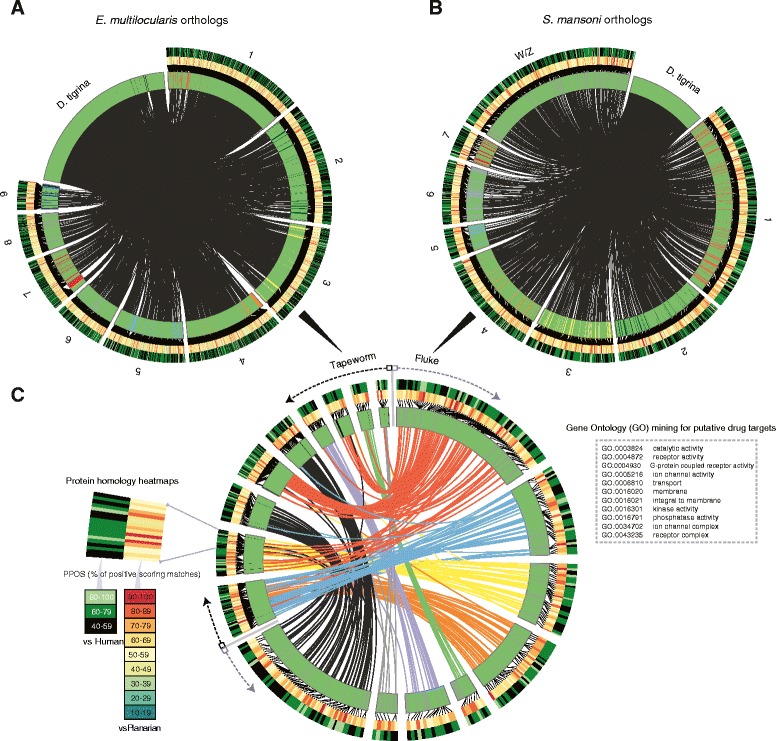
**Mapping of orthology relationships between** ***G. tigrina*** **and the genomes of pathogenic flatworms. A** and **B)** Circos diagram depicting ortholog pairs between arbitrarily arranged *G. tigrina* transcriptome and the genomes of the tapeworm *E. multilocularis* and the blood fluke *S. mansoni*. **C)** Ideograms are shown for *E. multilocularis* and *S. mansoni* chromosomes. Physical ortholog links reveal synteny between these parasites for putative drug targets. Links are shown only where there exists a *G. tigrina* ortholog. Drug targets were extracted by mining the *G. tigrina* predicted proteome for GO terms displayed in the box on the right. The inner heat map shows the percent similarity (ppos) between parasite and planarian ortholog protein pairs, and the outer heat map shows similarity between a given parasite protein and its nearest human homolog (RefSeq).

To better visualize these relationships, a Circos [36] diagram was created that mapped the chromosomal arrangements of orthologous genes for the selected parasites (Figure 4). Given that a stand-alone transcriptome lacks this spatial information, *G. tigrina* transcripts were arbitrarily ordered to allow for the mapping of planarian transcripts to the parasite genomes. Figures 4A and 4B show pairwise individual sequence comparisons between *G. tigrina* and the two parasites. The idiograms highlight the genomic locations of identified parasite orthologs, and are surrounded by heat maps that display the percent sequence similarity shared for each planarian-parasite ortholog pair, as well as for each parasite sequence and its nearest-matching human homolog, identified with BLASTp searches against the RefSeq human proteome.

### Drug target prioritization

Ortholog groups with high sequence conservation through the phylum represent potential broad-spectrum therapeutic targets, and these can plausibly be interrogated using *G. tigrina* as a more tractable free-living model. Ideally, we want to prioritize planarian protein targets that share very high sequence similarity with both fluke and tapeworm homologs, and that exhibit lower levels of sequence similarity with any identifiable host (human) proteins. Figure 4C applies this selection logic towards exploitation of the available sequence data. Here, links between *S. mansoni* and *E. multilocularis* reveal synteny for those orthologous gene pairs that share a highly similar *G. tigrina* ortholog and which represent useful anthelmintic targets. The final links are restricted to a set of 441 putative drug targets, filtered from the initial set of 2,693 ortholog groups (Additional file 2).

To estimate the druggability of protein targets, we utilized GO annotations that are most often associated with established drug target classes (http://www.ebi.ac.uk/thorntonsrv/databases/cgi-bin/drugport/GetPage.pl?template=goanal.html). Highly-associated GO terms, manually supplemented, were collected into a list and used to extract specific sequences from the annotated ortholog dataset. Within this set, we looked to identify a handful of targets as proof of concept for our model paradigm. Specifically, three targets were chosen that showed high sequence similarity between free-living and parasitic flatworms and that we could potentially pharmacologically manipulate or inhibit with commercially available chemicals. These targets were actin-related protein complex 2/3 subunit 2 (ARPC2), succinate dehydrogenase (SDH1), and NADH dehydrogenase (ubiquinone) flavoprotein 2 (NDUFV2).

### Comparative chemical screen of targets

To carry out a comparative first-pass phenotypic screen, chemical inhibitors for each target were used to treat newly transformed *S. mansoni* schistosomula and planaria across a range of concentrations. It should be noted that these chemical inhibitors have been shown to act on mammalian proteins, and the specificity of each interaction is therefore unknown. However, there is significant sequence conservation between protein domains in these proof of concept targets and their human homologs (Additional file 3), suggesting a high likelihood of a conserved mode of action. For example, *G. tigrina* ARPC2 shares 58% sequence identity with its human counterpart. While it can be hypothesized that CK-666, which locks this complex in an inactive state [49], performs this action in flatworms as well, it cannot be necessarily inferred.

**Figure 5:**
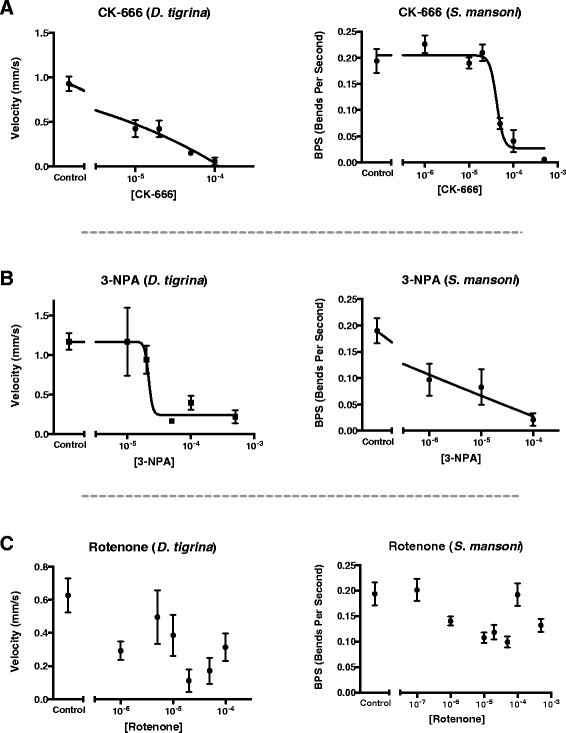
**Comparative effects of pharmacological inhibition on motility. A-C)** Pharmacological inhibition of three putative targets via available chemical inhibitors leads to correlative motility phenotype in free-living (*G. tigrina*) and parasitic flatworms (*S. mansoni*). Chemical inhibitors of ARPC2 **(A)** and SDH1 **(B)** caused a dose-dependent decrease in motility in both species as measured by contractions per second (schistosomula) or millimeters of translational movement per second (planaria). Chemical inhibitor of NDUFV2 **(C)** did not have any dose-dependent effects in either organism. Nonlinear regression is fit to a four-parameter variable slope model; log(inhibitor) vs. response. Bars represent SEM from the combination of two experiments.

Motility phenotypes were measured in terms of body contractions (bends per second) for *S. mansoni* and average velocity (mm per second) for *G. tigrina*. As shown in Figure 5 the dose response curves for each chemical elicited a similar phenotypic response profile for both *S. mansoni* and *G. tigrina*. CK-666 (ARPC2 inhibitor) and 3-nitropropionic acid (3-NPA; SDH1 inhibitor) caused dose-dependent decreases in motility in both worms. In contrast, rotenone (NADH dehydrogenase inhibitor) did not alter either worm’s motility. This further evidences the notion that pharmacological manipulation of highly conserved flatworm molecules in planaria can be predictive of phenotypic outcomes in schistosomes. One target that brought about a phenotypic effect in the pharmacological screen, ARPC2, and one that showed no apparent effect, NDUFV2, were then further examined with RNA interference (RNAi).

### RNAi in planaria is predictive of phenotypes in parasites

**Figure 6:**
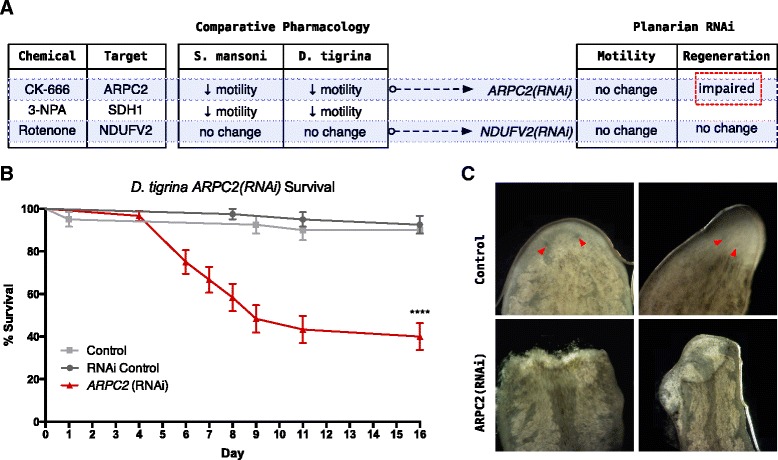
**RNAi phenotypes correlate to pharmacology screen. A)** Comparative pharmacology led to the selection of ARPC2 (increased motility) and NDUFV2 (no noticeable phenotype) as proof of principle targets for RNAi-mediated knock-down. RNAi of both targets did not bring about any significant changes in planarian motility. However, ARPC2 suppression was lethal to regenerating *G. tigrina*. These observations correlate to the pharmacology screen where no phenotype was observed with application of NDUFV2 inhibitor, but stark motility phenotypes were observed with ARPC2 chemical inhibitor. **B)** Survival curves show significantly decreased (P < 0.0001; Log-rank Mantel-Cox test) rates of survival for ARPC2(RNAi) cut worms in comparison to control cut worms. **C)** Prior to death, caudal fragments of ARPC2(RNAi) cut worms showed impaired sealing of the initial wound and improper blastema formation. Proper eye spot formation can be observed in control worms (red arrows) and is absent in the ARPC2 suppressed worms. These experiments were carried out in duplicate, with at least 10 whole planaria used for motility assays and 20 planarian halves used to monitor regeneration.

Complementary to the pharmacological screen, the expression of ARPC2 and NDUFV2 was suppressed using RNAi. After three dsRNA feedings dispersed over a 7 day timeline, semi-quantitative PCR was used to confirm near-complete transcript knock-down (Additional file 4). Phenotypic analyses were carried out on *G. tigrina* by monitoring motility and regeneration as commonly assayed outcomes of gene suppression in planaria. Motility was not significantly altered for either experimental group (Figure 6A), although this might be expected for NDUFV2 considering chemical inhibition resulted in no motility phenotype.

To assay regeneration, worms were bisected above the pharynx and each half was maintained in a separate well. Cephalic and caudal regeneration was observed over the course of 2–3 weeks. No developmental phenotype was observed for Dtig-NDUFV2(RNAi) worms, however, Dtig-ARPC2(RNAi) worms showed aberrant regeneration in comparison to control worms, consisting of a range of specific outcomes that included stalled or slowed regeneration, blastema malformation, an inability to seal the wound, and eventual death (Figures 6B and 6C). In these assays, readily identifiable phenotypes were observed in planaria that were predictive of visible phenotypes in the comparative chemical screen.

This proof of principle was limited to a handful of putative targets with commercially available inhibitors, but the pipeline can readily be scaled to larger numbers of targets. In the proposed scheme, highly conserved planarian-parasite orthologs are first interrogated with RNAi in planaria. The detection of phenotypes relating to planarian motility, morphology, or regeneration can serve as an efficient and high- throughput filter for target druggability in flatworm parasites. Parasite orthologs can then be investigated using available techniques such as RNAi and heterologous expression for drug target validation and functional characterization. This approach sidesteps the often prohibitive costs and technical challenges of carrying out large high-throughput screens in transient parasite life stages.

## Conclusion

This work further promotes the adoption of planaria, and in particular *Girardia tigrina*, as a model screening organism for candidate drug targets in parasites. We provide a high-coverage annotated *de novo* transcriptome as a substrate for such efforts. The Assemblies, CDS predictions and annotations are available for download (http://goo.gl/BZU87d). The identification of ortholog groups that extend to planaria, blood flukes, and tapeworms, allows for the rational prioritization of likely broad-spectrum drug targets that can be readily screened in *G. tigrina*. We outline a pathway for the high-throughput evaluation of putative drug targets in planaria as a prelude to validation and more extensive characterization in parasitic flatworms. We further show how such screens can be predictive of biological phenotypes in parasites.

This study builds on other recent studies that have shown the utility of the planarian system in understanding parasite biology. For example, the antischistosomal praziquantel has been shown to lead to changes in planarian regenerative polarity through the action of voltage-operated calcium channel (VOCC) β subunits [50]. In this conserved signaling pathway, regenerative polarity in planaria acts as a phenotypic correlate of drug efficacy and worm paralysis in schistosomes. This was followed by a more comprehensive investigation of the phenotypic correlates of manipulating signal transduction pathways in the planarian *D. japonica* and the parasite *S. mansoni*, as a predictive tool for the discovery of antischistosomal agents [51]. The striking identification of adult stem cells in *S. mansoni* that resemble planarian neoblasts further strengthens the notion of fundamental biological conservation between free-living and parasitic flatworms [52]. This annotated *G. tigrina* sequence resource, along with the orthology-based prioritization of putative drug targets, can help catalyze low cost and scalable *in vivo* pipelines for anthelmintic drug discovery.

## Additional files

Additional file 1:
**Differential Expression.** PDF file containing clustered heatmaps and graphs for differentially expressed transcripts. Pages 1–2 show differential expression for control vs. cut animals. Pages 3–4 show differential expression for cut (no serotonin) vs. cut (serotonin) animals. Additional file 2:
**Druggable ortholog list.** Excel spreadsheet containing core ortholog sequence set organized by species and ID. Additional file 3:
**Flatworm-Human target homology.** PDF file containing sequence alignments for the three putative drug targets probed in this study. Each *G. tigrina* target sequence was aligned against its nearest *S. mansoni*, *E. multilocularis*, and human homolog. Additional file 4:
**Semi-quantitative RT-PCR of RNAi experiments.** PDF file containing images of representative semi-quantitative RT-PCR gels. The first four non-ladder lanes are RNAi worms and the last two lanes are negative controls. The bottom bands are 18S Ribosomal RNA reference reference (300 bp), and the top bands are ARPC2 or NDUFV2 amplicons (600 bp).

## References

[1] WHO (2012). Schistosomiasis: population requiring preventive chemotherapy and number of people treated in 2010. Wkly Epidemiol. Rec..

[2] van der Werf MJ, de Vlas SJ, Brooker S, Looman CWN, Nagelkerke NJD, Habbema JDF, Engels D (2003). Quantification of clinical morbidity associated with schistosome infection in sub-Saharan Africa. Acta Trop..

[3] Murray CJL, Vos T, Lozano R, Naghavi M, Flaxman AD, Michaud C (2012). Disability-adjusted life years (DALYs) for 291 diseases and injuries in 21 regions, 1990–2010: a systematic analysis for the Global Burden of Disease Study 2010. Lancet.

[4] Torreele E, Usdin M, Chirac P, Iyer BJK, Adams ER, Klatser PR: Priority medicines for Europe and the world: a public health approach to innovation. 2004

[5] Wang W, Wang L, Liang Y-S (2012). Susceptibility or resistance of praziquantel in human schistosomiasis: a review. Parasitol. Res..

[6] Gilbert IH (2013). Drug discovery for neglected diseases: molecular target-based and phenotypic approaches. J. Med. Chem..

[7] Chen B, Wen J-F (2011). The adaptive evolution divergence of triosephosphate isomerases between parasitic and free-living flatworms and the discovery of a potential universal target against flatworm parasites. Parasitol. Res..

[8] Scimone M, Kravarik K, Lapan S, Reddien P (2014). Neoblast Specialization in Regeneration of the Planarian Schmidtea mediterranea. Stem Cell Rep..

[9] Zhang J, Yuan Z, Zheng M, Sun Y, Wang Y, Yang S (2013). Effects of N,N-dimethylformamide on behaviour and regeneration of planarian Dugesia japonica. Toxicol. Ind. Health.

[10] Evans DJ, Owlarn S, Tejada Romero B, Chen C, Aboobaker A (2011). Combining classical and molecular approaches elaborates on the complexity of mechanisms underpinning anterior regeneration. PLoS One.

[11] Reddien PW, Bermange AL, Murfitt KJ, Jennings JR, Sanchez Alvarado A (2005). Identification of genes needed for regeneration, stem cell function, and tissue homeostasis by systematic gene perturbation in planaria. Dev. Cell.

[12] Collins JJ, Hou X, Romanova EV, Lambrus BG, Miller CM, Saberi A, Sweedler JV, Newmark P (2010). Genome-wide analyses reveal a role for peptide hormones in planarian germline development. PLoS Biol..

[13] Gilleard JS (2004). The use of Caenorhabditis elegans in parasitic nematode research. Parasitology.

[14] Robb SMC, Ross E, Sa A (2008). SmedGD : the Schmidtea mediterranea genome database. Nucleic Acids Res..

[15] Abril JF, Cebri’a F, Rodrıguez-Esteban G, Horn T, Fraguas S, Calvo B, Bartscherer K, Sal’o E (2010). Smed454 dataset: unravelling the transcriptome of Schmidtea mediterranea. BMC Genomics.

[16] Galloni M (2012). Global irradiation effects, stem cell genes and rare transcripts in the planarian transcriptome. Int J Dev Biol.

[17] Resch AM, Palakodeti D, Lu Y-C, Horowitz M, Graveley BR (2012). Transcriptome analysis reveals strain-specific and conserved stemness genes in Schmidtea mediterranea. PLoS One.

[18] Solana J, Kao D, Mihaylova Y, Jaber-Hijazi F, Malla S, Wilson R, Aboobaker A (2012). Defining the molecular profile of planarian pluripotent stem cells using a combinatorial RNAseq, RNA interference and irradiation approach. Genome Biol..

[19] Nishimura O, Hirao Y, Tarui H, Agata K (2012). Comparative transcriptome analysis between planarian Dugesia japonica and other platyhelminth species. BMC Genomics.

[20] Garcia-Fernandez J, Ram J, Mar-any G, Mun AM (1995). High Copy Number of Highly Similar mariner-like Transposons in Planarian ( Platyhelminthe ): Evidence for a Trans-Phyla Horizontal Transfer. Mol Biol Evol.

[21] Kreshchenko ND (2008). Functions of flatworm neuropeptides NPF, GYIRF and FMRF in course of pharyngeal regeneration of anterior body fragments of planarian, *Girardia* tigrina. Acta Biol. Hung..

[22] Pag’an OR, Deats S, Baker D, Montgomery E, Wilk G, Tenaglia M, Semon J (2013). Planarians require an intact brain to behaviorally react to cocaine, but not to react to nicotine. Neuroscience.

[23] Ramakrishnan L, Amatya C, DeSaer CJ, Dalhoff Z, Eggerichs MR (2014). Galantamine reverses scopolamine-induced behavioral alterations in Dugesia tigrina. Invert Neurosci.

[24] Bolger AM, Lohse M, Usadel B.: Trimmomatic: a flexible trimmer for Illumina sequence data. Bioinformatics. 30(15):2114-20. doi:10.1093/bioinformatics/btu170 10.1093/bioinformatics/btu170 PMC4103590 24695404

[25] Magoč T, Salzberg SL (2011). FLASH: fast length adjustment of short reads to improve genome assemblies. Bioinformatics.

[26] Grabherr M, Haas B, Yassour M (2011). Trinity: reconstructing a full-length transcriptome without a genome from RNA-Seq data. Nature.

[27] Zerbino DR, Birney E (2008). Velvet: algorithms for de novo short read assembly using de Bruijn graphs. Genome Res..

[28] Schulz MH, Zerbino DR, Vingron M, Birney E (2012). Oases: robust de novo RNA-seq assembly across the dynamic range of expression levels. Bioinformatics.

[29] Hornik, K.: The R FAQ (2014). http://cran.r-project.org/doc/FAQ/R-FAQ.html

[30] Langmead B, Trapnell C, Pop M, Salzberg SL (2009). Ultrafast and memory-efficient alignment of short DNA sequences to the human genome. Genome Biol..

[31] Li B, Dewey CN (2011). RSEM: accurate transcript quantification from RNA-Seq data with or without a reference genome. BMC Bioinformatics.

[32] Li W, Godzik A (2006). Cd-hit: a fast program for clustering and comparing large sets of protein or nucleotide sequences. Bioinformatics.

[33] Conesa A, G’otz S, Garc’ıa-G’omez JM, Terol J, Tal’on M, Robles M (2005). Blast2GO: a universal tool for annotation, visualization and analysis in functional genomics research. Bioinformatics.

[34] Lechner M, Findeiss S, Steiner L, Marz M, Stadler PF, Prohaska SJ (2011). Proteinortho: detection of (co-)orthologs in large-scale analysis. BMC Bioinformatics.

[35] Logan-Klumpler FJ, De Silva N, Boehme U, Rogers MB, Velarde G, McQuillan J, Carver T, Aslett M, Olsen C, Subramanian S, Phan I, Farris C, Mitra S, Ramasamy G, Wang H, Tivey A, Jackson A, Houston R, Parkhill J, Holden M, Harb OS, Brunk BP, Myler PJ, Roos D, Carrington M, Smith DF, Hertz-Fowler C, Berriman M (2012). GeneDB–an annotation database for pathogens. Nucleic Acids Res..

[36] Krzywinski M, Schein J, Birol I, Connors J, Gascoyne R, Horsman D, Jones SJ, Marra M (2009). Circos: an information aesthetic for comparative genomics. Genome Res..

[37] Camacho C, Coulouris G, Avagyan V, Ma N, Papadopoulos J, Bealer K, Madden TL (2009). BLAST+: architecture and applications. BMC Bioinformatics.

[38] Pruitt KD, Brown GR, Hiatt SM, Thibaud-Nissen F, Astashyn A, Ermolaeva O, Farrell CM, Hart J, Landrum MJ, McGarvey KM, Murphy MR, Database issue (2014). RefSeq: an update on mammalian reference sequences. Nucleic Acids Res.

[39] Rask-Andersen M, Alm’en MS, Schïoth HB (2011). Trends in the exploitation of novel drug targets. Nat. Rev. Drug Discov..

[40] Milligan JN, Jolly ER (2011). Cercarial transformation and in vitro cultivation of Schistosoma mansoni schistosomules. J Vis Exp.

[41] Schneider CA, Rasband WS, Eliceiri KW (2012). NIH Image to ImageJ: 25 years of image analysis. Nat Methods.

[42] Noldus LP, Spink AJ, Tegelenbosch RA (2001). EthoVision: a versatile video tracking system for automation of behavioral experiments. Behav Res Methods Instruments Comp.

[43] Koressaar T, Remm M (2007). Enhancements and modifications of primer design program Primer3. Bioinformatics.

[44] Rouhana L, Weiss J, Forsthoefel DJ, Lee H, King RS, Inoue T, Shibata N, Agata K, Newmark P (2013). RNA interference by feeding in vitro-synthesized double-stranded RNA to planarians: methodology and dynamics. Dev Dyn.

[45] Kao D, Felix D, Aboobaker A (2013). The planarian regeneration transcriptome reveals a shared but temporally shifted regulatory program between opposing head and tail scenarios. BMC Genomics.

[46] Patocka N, Ribeiro P (2013). The functional role of a serotonin transporter in Schistosoma mansoni elucidated through immunolocalization and RNA interference (RNAi). Mol Biochem Parasitol.

[47] Jones P, Binns D, Chang H-Y, Fraser M, Li W, McAnulla C, McWilliam H, Maslen J, Mitchell A, Nuka G, Pesseat S, Quinn AF, Sangrador-Vegas A, Scheremetjew M, Yong S-Y, Lopez R, Hunter S (2014). InterProScan 5: genome-scale protein function classification. Bioinformatics (Oxford, England).

[48] Kanehisa M, Goto S, Sato Y, Kawashima M, Furumichi M, Tanabe M (2014). Data, information, knowledge and principle: back to metabolism in KEGG. Nucleic Acids Res..

[49] Hetrick B, Han MS, Helgeson LA, Nolen BJ (2013). Small molecules CK-666 and CK-869 inhibit actin-related protein 2/3 complex by blocking an activating conformational change. Chem Biol.

[50] Nogi T, Zhang D, Chan JD, Marchant JS (2009). A novel biological activity of praziquantel requiring voltage-operated Ca2+ channel beta subunits: subversion of flatworm regenerative polarity. PLoS Negl. Trop. Dis..

[51] Crowther GJ, Shanmugam D, Carmona SJ, Doyle MA, Hertz-Fowler C, Berriman M, Nwaka S (2010). Identification of attractive drug targets in neglected-disease pathogens using an in silico approach. PLoS Neglected Trop Dis.

[52] Wang B, Collins JJ, Newmark PA (2013). Functional genomic characterization of neoblast-like stem cells in larval Schistosoma mansoni. eLife.

