# Supplementary figures and images for: Functional analysis of Girardia tigrina transcriptome seeds pipeline for anthelmintic target discovery
Source: Parasit Vectors. 2015 Jan 20;8:34. doi: 10.1186/s13071-014-0622-3 (PMC4304616; doi:10.1186/s13071-014-0622-3)

Color Key

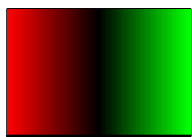

-2 0 2

Value

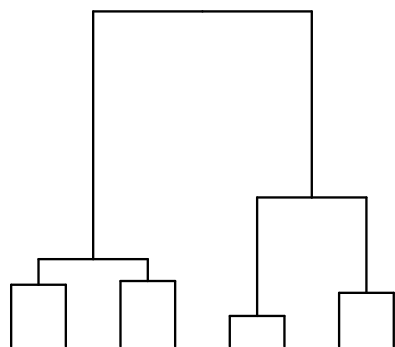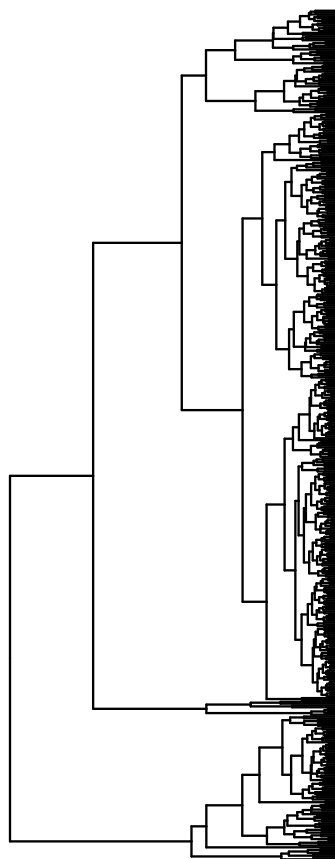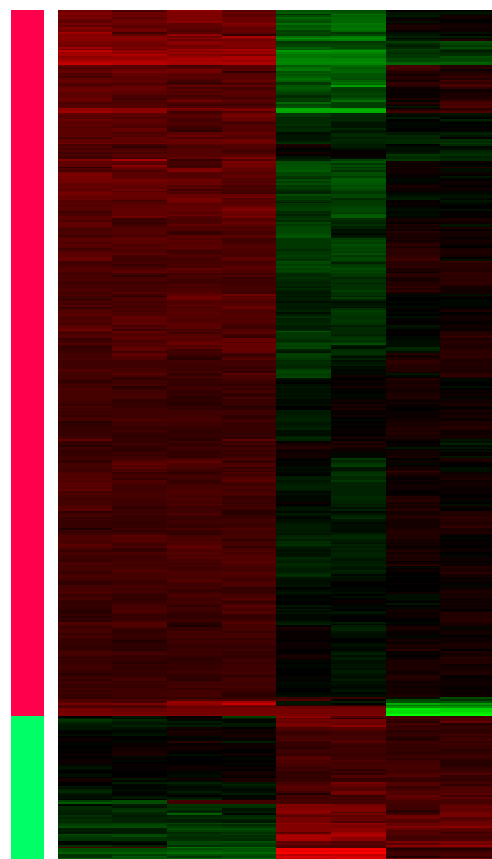

Cut1  
Cut2  
Ser1  
Ser2  
Con3  
Con4  
Con1  
Con2

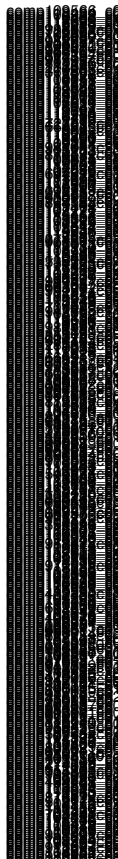

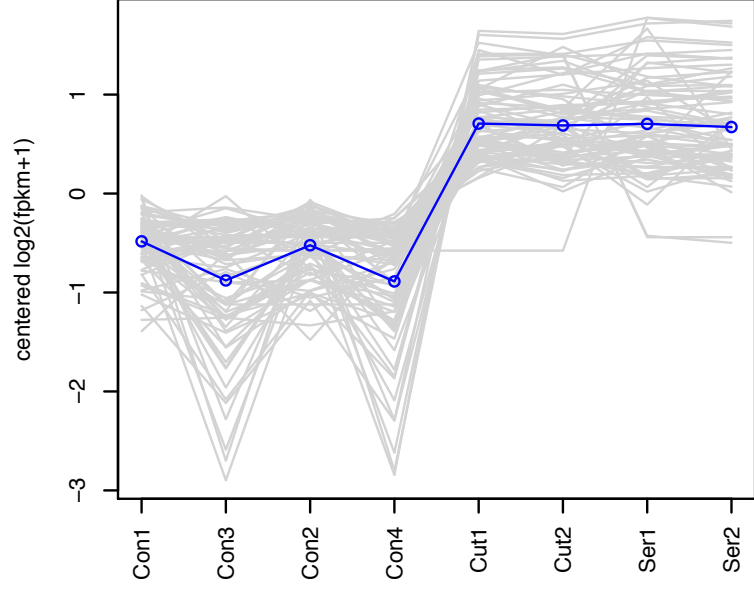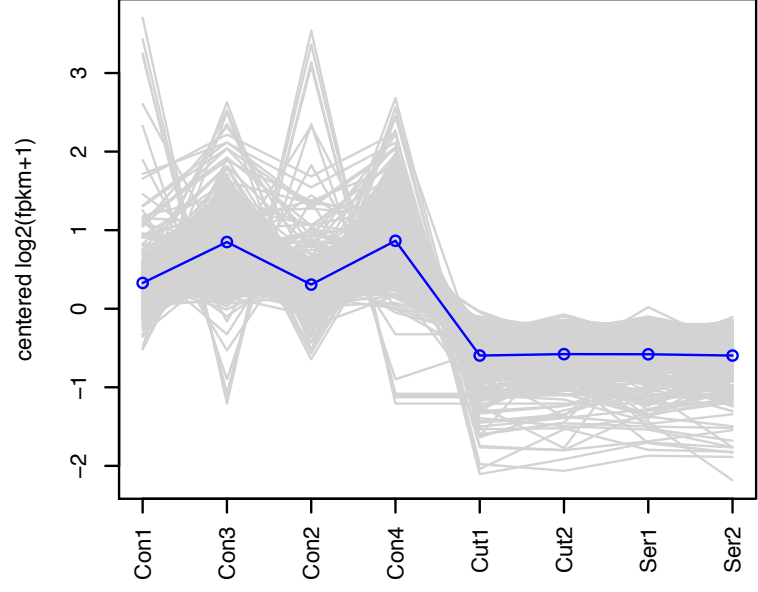

# Color Key

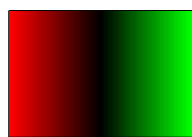

-1 0 1

Value

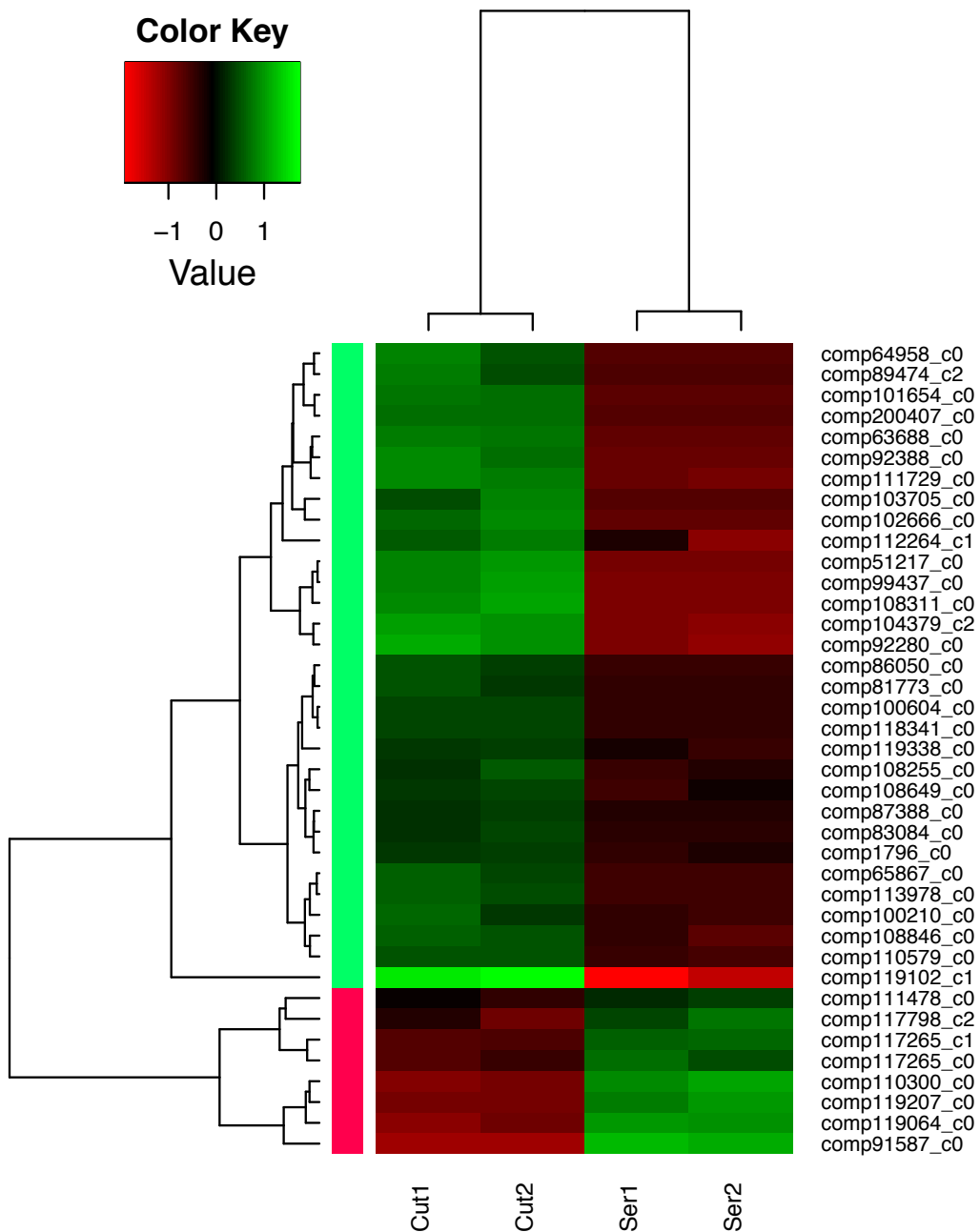

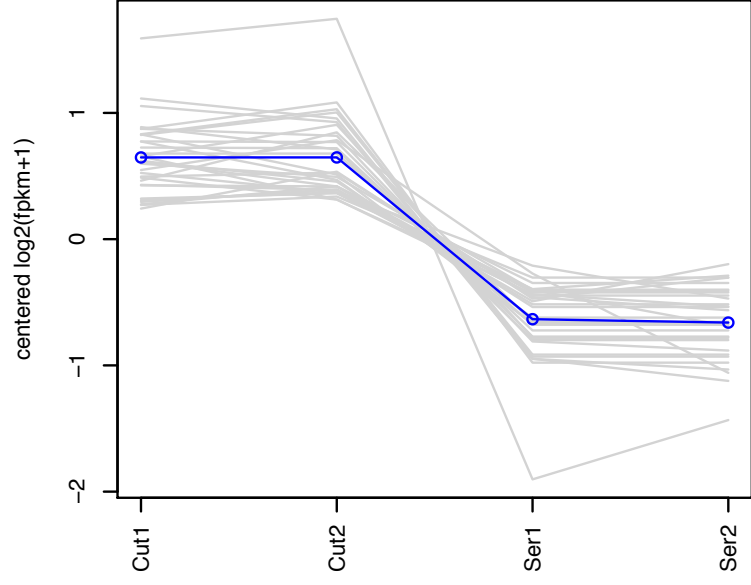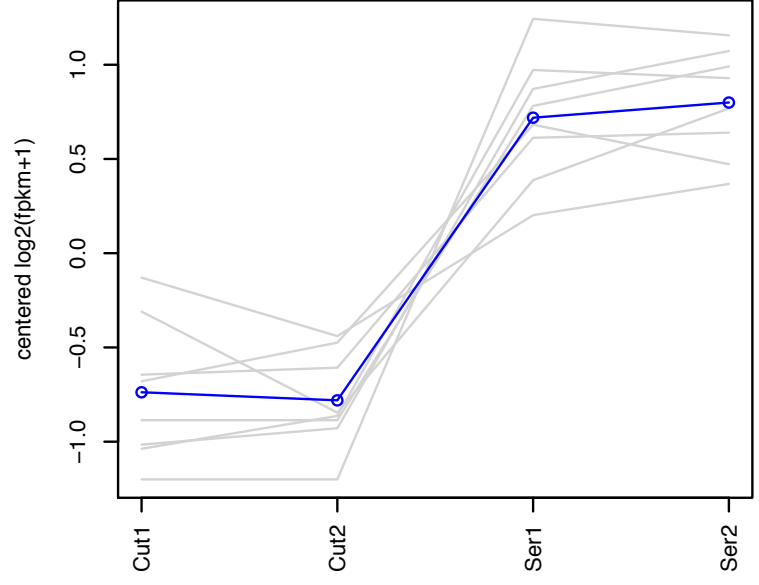

Supplement: Additional file 1: — Differential Expression. PDF file containing clustered heatmaps and graphs for differentially expressed transcripts. Pages 1–2 show differential expression for control vs. cut animals. Pages 3–4 show differential expression for cut (no serotonin) vs. cut (serotonin) animals. [file 13071_2014_622_MOESM1_ESM.pdf]

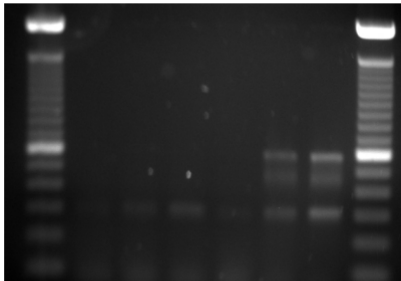

ARPC2 RNAi (4 RNAi + 2 controls)

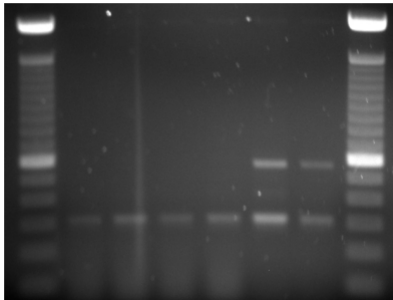

NDUFV2 RNAi (4 RNAi + 2 controls)

Supplement: Additional file 4: — Semi-quantitative RT-PCR of RNAi experiments. PDF file containing images of representative semi-quantitative RT-PCR gels. The first four non-ladder lanes are RNAi worms and the last two lanes are negative controls. The bottom bands are 18S Ribosomal RNA reference reference (300 bp), and the top bands are ARPC2 or NDUFV2 amplicons (600 bp). [file 13071_2014_622_MOESM4_ESM.pdf]
