# Supplementary material for: Functional analysis of Girardia tigrina transcriptome seeds pipeline for anthelmintic target discovery
Source: Parasit Vectors. 2015 Jan 20;8:34. doi: 10.1186/s13071-014-0622-3 (PMC4304616; doi:10.1186/s13071-014-0622-3)
Supplement: Additional file 3: — Flatworm-Human target homology. PDF file containing sequence alignments for the three putative drug targets probed in this study. Each G. tigrina target sequence was aligned against its nearest S. mansoni, E. multilocularis, and human homolog. [file 13071_2014_622_MOESM3_ESM.pdf]

```
                       1         10        20        30        40        50
Dtig_m.24616           ..LLEYFNRGVKEVLLQKFTAAKAGNKIEKIKQCLADFDGAIYKINNEK.DKNIIYLSIC
Hsap_NP_690601.1       MILLEVNNRIIEETLALKFENAAAGNKPEAVEVTFADFDGVLYHISNPNGDKTKVMVSIS
Sman_Smp_010690        MIILEVCNRAVEEILVTRFEQAKEESRYDKIDYTVADFDSIVYTISNPNNDKKKILVSIF
Emul_000305600.1..pep  MIFLEFHNSAVEDLLLTRFSNAKAGFKFEKIDYTVADFDRVLYRIHNPEGDKSKLLVSLL

                       60        70        80        90        100       110
Dtig_m.24616           LKFFKQLQEYDADKVLKREYGDRLCSPPEPGCNVTIQFDLTKLPDDIEELAHNAALLKRN
Hsap_NP_690601.1       LKFYKELQAHGADELLKRVYGSFLVNP.ESGYNVSLLYDLENLPASKDSIVHQAGMLKRN
Sman_Smp_010690        IKFFHELKEHGVNEVLAREYGDFLSSAE.PNQSVTLCIDLEKLPSDHNQLAHKCGLLKRN
Emul_000305600.1..pep  VNFFDELKEYDVEGLLRREYGAYILDEPYPGYSVTLCFDLQNVPEDCEVVARHVAMLKRN

                       120       130       140       150       160       170
Dtig_m.24616           CFASVFETFFDYQESGNIAK...QATINYRDDETMYVKAMEDRVTVIFSTVFKDSDDIII
Hsap_NP_690601.1       CFASVFEKYFQFQEEGKEGEN..RAVIHYRDDETMYVESKKDRVTVVFSTVFKDDDDVVI
Sman_Smp_010690        CMAAVFEKFFEYQIKSNADSGSKRAVIHYRDDETMYVQALADRVTVIFSTTFKDPDDLLI
Emul_000305600.1..pep  CFAAVFEPFFLLQALADEPIISKRAVIHYSPDEAMYVQALEDRITVIFSTVFKDADDVVI

                       180       190       200       210       220       230
Dtig_m.24616           GKVFIQELTEARRRVDRAPQVLYSPKEPPIELKGTKAAVGENVAYITFVLFNRHFATPAA
Hsap_NP_690601.1       GKVFMQEFKEGRRASHTAPQVLFSHREPPLELKDTDAAVGDNIGYITFVLFPRHTN.ASA
Sman_Smp_010690        GKVFMQEFTEVRRRLDRAPQVLYSHRVPPKELQGTDAVISDSVAYITFVLFPRHLSTESA
Emul_000305600.1..pep  GKIFMQEFTEVRRRYDRAPQVLYSHREPPAELRDTDAVGGDNRAYITFVLFPRHLAPHAA

                       240       250       260       270       280
Dtig_m.24616           RKNSIDLIHTFRNYLHYHIKCSKAHIHMRMRAKSGNFLKLLKRAKP............EI
Hsap_NP_690601.1       RDNTINLIHTFRDYLHYHIKCSKAYIHTRMRAKTSDFLKVLNRARP............DA
Sman_Smp_010690        RNSTIDLIQTLRNYLHYHIKCSKAYMQMRMRAKTVEFLKVLNRAH.............I
Emul_000305600.1..pep  DRS.INLIHTVRNYLHYHIKCAKGYLQLRMRAKTSEFIKILNRAVPHQQQQQQQQQLNDG

                       290       300       310       320
Dtig_m.24616           SASGSGCEAVDFRGVTD.......LGIEGLNLGSGDSLSQKKTFTGRTFLPQS.......
Hsap_NP_690601.1       EKK....................................EMKTITGKTFSSR........
Sman_Smp_010690        EHSTNTVINTNITTVSGEVAPISPLMNKIGGIQSDN........MGRTMRTG........
Emul_000305600.1..pep  EESAEPAVFVHALGLESSRSQIHRQPANRRGLASGNAFSSENMLMPSTLSQSDFACLQKF

Dtig_m.24616           ................................
Hsap_NP_690601.1       ................................
Sman_Smp_010690        ................................
Emul_000305600.1..pep  EDSEDEREEEEDGVLSAPLMANSIPPSPCFDA
```

```
                    1        10          20                 30        40        50
Sman_Smp_069770     ....MALRTFGSVSLKLLCRSDGSLFRNF.....GRQSSHLFVHRETRENNSNTPFEFSA
Dtig_m.6038         .....MFRNF....YQNIYRG.IQLKR.......RCLSDKLFTHRDTPDNNANTLFEFNS
Emul_001114700      ..MGLNIANQCFWSLRMLSASKVSLLNTLPLKAFRSFSTALFWHRDTAINNRDVLFEFTP
Hsap_NP_066552.2    MFFSAALRARAAGLTAHWGRHVRNLHKTVMQ...NGAGGALFVHRDTPENNPDTPFDFTP

                           60        70        80        90       100       110
Sman_Smp_069770     ENKKRLDVIISNYPPAHKAAAIIPALDLAQRQHGWLPISAMNKVAEILNVPQMRVYEVAT
Dtig_m.6038         ENLKRIETIKSNYPDGHQNAALIPVLDLAQRQHGWLPISAMNKVAEILDMPRMRVYEVAT
Emul_001114700      ENLRRLEELKTHYPVGYHASLIMPALDIAQRQNGWLPISAMSKIAEYLGVPEMRVFEVAT
Hsap_NP_066552.2    ENYKRIEAIVKNYPEGHKAAAVLPVLDLAQRQNGWLPISAMNKVAEVLQVPPMRVYEVAT

                          120       130       140       150       160       170
Sman_Smp_069770     FYTMFNREPVGKYHIQICTTTPCMLGGVGSEVILNALKKNLGIEPGQTTPDKMFTLTEVE
Dtig_m.6038         FYTMFNRDPVGRYHVQLCTCSPCILGGVGSDIILETIKKHLGIKPGETTSDKLFTLSEVE
Emul_001114700      FYTMFNRNPVGKYHVQLCTTTPCMLGGVGSDVILETIEKTLGIKTGETTPDKLFTISQVE
Hsap_NP_066552.2    FYTMYNRKPVGKYHIQVCTTTPCMLR..NSDSILEAIQKKLGIKVGETTPDKLFTLIEVE

                          180       190       200       210       220       230
Sman_Smp_069770     CLGACVNAPMMQINDDYYEDLTAEDTIRILEEIKAGKKPKPGPQSGQGGRFASEPKGGLT
Dtig_m.6038         CLGACVNAPMMAVNDDYYEDLTVDDTVRILNDFKANKRPKPGPQSGQSNRFACEPKGKLT
Emul_001114700      CLGACVNAPMMQINDDYYEDLTPDDVVRILSDLKAGKKPKHGPQSGQGHRCACEPKGGFT
Hsap_NP_066552.2    CLGACVNAPMVQINDNYYEDLTAKDIEEIIDELKAGKIPKPGPRSG...RFSCEPAGGLT

                          240
Sman_Smp_069770     SLNTEPKSPGFKVRSDL......
Dtig_m.6038         SLTEEPKGPGFKVRSDL......
Emul_001114700      SLNTPPVPPDFKLQDALKYTVDI
Hsap_NP_066552.2    SLTEPPKGPGFGVQAGL......
```

```
                   1        10        20        30        40        50
Emul_000422600     .MEKPPNCQFSCTFMAFLVRASFASFAARLG.....CLPTFAGASRHVSTVGKDYTIIDH
Sman_Smp_061230    ......MVHLALSLNAIRKATKLNYLTN........IYQSFK..CRNLSYT.SGYTIIDH
Dtig_m.21611       ......MSRLLCNLKRVYEFSGKKVGISR........FPTRR..LLSSTQISKDYTIVDH
Hsap_NP_004159.2   MSGVRGLSRLLSARRLALAKAWPTVLQTGTRGFHFTVDGNKRASAKVSDSISAQYPVVDH

                     60        70        80        90        100       110
Emul_000422600     TYDAVVVGAGGAGLRAAFGLANEGFKTACVTKLFPTRSHTVAAQGGINAALGNMEQDHWK
Sman_Smp_061230    SFDAVVVGAGGAGLRAGFGLANEGFKTAIITKLFPTRSHTVAAQGGINAALGNMEPDDWR
Dtig_m.21611       TYDAVVVGAGGAGLRAAFGLANSGFKTACITKLFPTRSHTVAAQGGINAALGNMEKDDWR
Hsap_NP_004159.2   EFDAVVVGAGGAGLRAAFGLSEAGFNTACVTKLFPTRSHTVAAQGGINAALGNMEEDNWR

                     120       130       140       150       160       170
Emul_000422600     FHMFDTVKGSDWLGDQDAIHYMCEEAPKAVIELENYGMPFSRLENGKIYQRAFGGQSLHY
Sman_Smp_061230    FHMYDTVKGSDWLGDQDAIHYMCEEAPKAVIELENYGVPFSRLENGKIYQRAFGGQSIDY
Dtig_m.21611       YHMYDTVKGSDWLGDQDAIHYMCEEAPKTVIELENYGMPFSRLDNGMIYQRAFGGQAIDY
Hsap_NP_004159.2   WHFYDTVKGSDWLGDQDAIHYMTEQAPAAVVELENYGMPFSRTEDGKIYQRAFGGQSLKF

                     180       190       200       210       220       230
Emul_000422600     GKGGQAHRCCAVADRTGHSLLHTLYGRSLRYETDYFIEYFALDLLMENGVCRGINAMCLE
Sman_Smp_061230    GRGGQAHRCCAVADRTGHSLLHTLYGRSLRYDATYFIEYFVLDLLMENGECRGVIAVCLE
Dtig_m.21611       GKGGQAHRCCCVADRTGHSLLHTLYGRSLKYDTDYFIEYFAMDLLMEHGECRGVLALNLE
Hsap_NP_004159.2   GKGGQAHRCCCVADRTGHSLLHTLYGRSLRYDTSYFVEYFALDLLMENGECRGVIALCIE

                     240       250       260       270       280       290
Emul_000422600     DGTIHRFRAKNTILATGGYGRAYFSCTSAHTCTGDGTAMVTRAGLPNEDMEFVQFHPTGI
Sman_Smp_061230    DGTLHRFRSKNTVLATGGYGRTYFSCTSAHTCTGDGTAMVTRAGLPNQDMEFVQFHPTGI
Dtig_m.21611       DGTLHRFRAKNTVLATGGYGRTFFSCTSAHTCTGDGTAMISRAGLPNEDMEFIQFHPTGI
Hsap_NP_004159.2   DGSIHRIRAKNTVVATGGYGRTYFSCTSAHTSTGDGTAMITRAGLPCQDLEFVQFHPTGI

                     300       310       320       330       340       350
Emul_000422600     YGAGCLITEGCRGEGGYLINSEGERFMERYAPNAKDLASRDVVSRAMTIEIREGRGVGPR
Sman_Smp_061230    YGAGCLITEGCRGEGGYLINSKGERFMERYAPNAKDLASRDVVSRAMTIEIREGRGVGPR
Dtig_m.21611       YGAGCLITEGCRGEGGYLINSEGERFMERYAPKAKDLASRDVVSRSMTIEIREGRGVGPQ
Hsap_NP_004159.2   YGAGCLITEGCRGEGGILINSQGERFMERYAPVAKDLASRDVVSRSMTLEIREGRGCGPE

                     360       370       380       390       400       410
Emul_000422600     KDHCYLKLHHLPPDLLKSRLPGISETAEIFAGVDVTKEPVPVLPTVHYNMGGVPTNYKGQ
Sman_Smp_061230    KDHIFLQLSHLPADQLHSRLPGISETAKIFAGVDVTRDPIPVLPTVHYNMGGIPTNYKGQ
Dtig_m.21611       KDHIFLQLSHLPMELLKHRLPGISETAMIFAGVDVTRDPIPVLPTVHYNMGGIPTNYKGQ
Hsap_NP_004159.2   KDHVYLQLHHLPPEQLATRLPGISETAMIFAGVDVTKEPIPVLPTVHYNMGGIPTNYKGQ

                     420       430       440       450       460       470
Emul_000422600     VITYDAATGKDKIIPGLYAAGEVACASVHGANRLGANSLLDLVVFGRACALDIAEKCKPG
Sman_Smp_061230    VLAYDPVAKKGKVVPGLYAAGEAASASVHGANRLGANSLLDIVVFGRACALDIAAKNKPG
Dtig_m.21611       VIDYDPHTKKDKIVKGLYAAGEAACTSVHGANRLGANSLLDIVVFGRACALTIAEENKPG
Hsap_NP_004159.2   VLRH..VNGQDQIVPGLYACGEAACASVHGANRLGANSLLDLVVFGRACALSIEESCRPG

                     480       490       500       510       520       530
Emul_000422600     DFGPELHPETGMESVANIDKLRSGKGQFPVSDVRLEMQRTMQEHAAVFRDGPVLKAGVEK
Sman_Smp_061230    DAGPELKPDTGEASIANYEKLRTANGSYPIAQVRLDMQRTMQEYAAVFRDGPTLKEGCKK
Dtig_m.21611       EKQPDLSKNAGEQSIANLDKVRHANGSLSTAEIRLKMQKTNQEHAAVFRDGPVLQAGCKK
Hsap_NP_004159.2   DKVPPIKPNAGEESVMNLDKLRFADGSIRTSELRLSMQKSMQNHAAVFRVGSVLQEGCGK

                     540       550       560       570       580       590
Emul_000422600     MLKLYAAKYDNLKVSDKSLIWNSDLIEGLELQNLLINAVQTIVAAEARKESRGAHAREDF
Sman_Smp_061230    MYDLYASRMNDLKVSDRSKIWNSDLMEALELQNLMLNALQTIVGAEARKESRGAHAREDF
Dtig_m.21611       ILDIYRNDMQQLKLSDRSMIWNSDLVEALELQNLMINSLQTIYGAENRKESRGAHSREDF
Hsap_NP_004159.2   ISKLYG.DLKHLKTFDRGMVWNTDLVETLELQNLMLCALQTIYGAEARKESRGAHAREDY
```

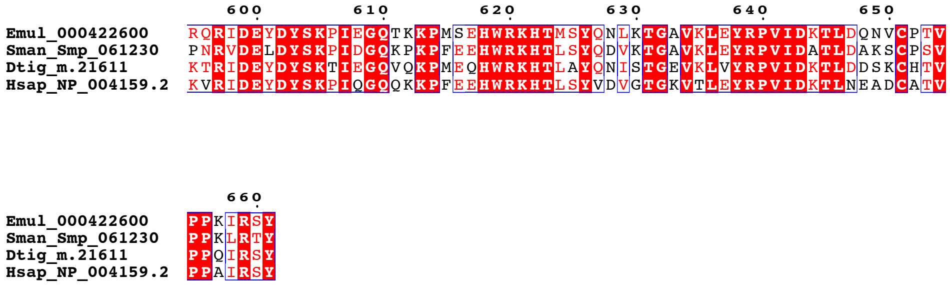
600 610 620 630 640 650
Emul_000422600 RQRIDEYDYSKPIEGQTKKPMSEHWRKHTMSYQNLKTGAVKLEYRPVIDKTLDQNVCPTV
Sman_Smp_061230 PNRVDELDYSKPIDGQKPKPFEEHWRKHTLSYQDVKTGAVKLEYRPVIDATLDAKSCPSV
Dtig_m.21611 KTRIDEYDYSKTIEGQVQKPMEQHWRKHTLAYQNISTGEVKLVYRPVIDKTLDDSKCHTV
Hsap_NP_004159.2 KVRIDEYDYSKPIQGQQKKPFEEHWRKHTLSYVDVGTGKVTLEYRPVIDKTLNEADCATV
660
Emul_000422600 PPKIRSY
Sman_Smp_061230 PPKLRTY
Dtig_m.21611 PPQIRSY
Hsap_NP_004159.2 PPAIRSY
